# Association of the newly proposed dietary index for gut microbiota and constipation: a cross-sectional study from NHANES

**DOI:** 10.3389/fnut.2025.1529373

**Published:** 2025-01-17

**Authors:** Zhuhui Zhang, Chunlu Bi, Runsheng Wu, Muwen Qu

**Affiliations:** ^1^Department of Anorectal Surgery, Guang’ Anmen Hospital, China Academy of Chinese Medical Sciences, Beijing, China; ^2^Department of Anorectal Surgery, Shenzhen Traditional Chinese Medicine Anorectal Hospital (Futian), Shenzhen, China; ^3^Department of Urology, Shenzhen Pingle Orthopedics Hospital, Shenzhen, Guangdong, China

**Keywords:** DI-GM, constipation, cross-sectional study, NHANES, bowel health

## Abstract

**Objective:**

The dietary index for gut microbiota. DI-GM is an innovative metric designed to capture the diversity of the gut microbiome, yet its association with constipation remains unstudied.

**Methods:**

In this cross-sectional study, 11,405 adults aged 20 and older were selected from the National Health and Nutrition Examination Survey 2005–2010 for the sample. Constipation was defined as fewer than three defecation frequencies per week using bowel health questionnaire (BHQ). Fewer than three bowel movements per week were considered as constipation by Bowel Health Questionnaire (BHQ). DI-GM was derived from dietary recall data, including avocado, broccoli, chickpeas, coffee, cranberries, fermented dairy, fiber, green tea, soybean and whole grains as beneficial elements, red meat, processed meat, refined grains, and high fat as detrimental components. Multivariable weighted logistic was employed to investigate the association of DI-GM with constipation. Secondary analyses included subgroup analyses, restricted cubic spline (RCS), and multiple imputation.

**Results:**

A higher DI-GM and beneficial gut microbiota score were associated with a lower prevalence of constipation (DI-GM: OR = 0.82, 95% CI = 0.75, 0.90; beneficial gut microbiota score: OR = 0.77, 95% CI = 0.67, 0.89). After grouping DI-GM, in the fully adjusted model, participants with DI-GM ≥ 6 were significantly negatively correlated with both the prevalence of constipation (OR = 0.48, 95% CI = 0.33, 0.71). RCS indicated a non-linear relationship between DI-GM and constipation. Subgroup analyses by age, sex and common complications showed no statistically significant interactions (*p* > 0.05).

**Conclusion:**

The newly proposed DI-GM was inversely related with the prevalence of constipation. When treating patients with constipation, it is necessary for clinicians to provide timely and effective dietary interventions incorporating the DI-GM for patients with constipation to avoid further deterioration of the condition.

## 1 Introduction

Constipation is defined by infrequent bowel movements, straining during defecation, and a sensation of incomplete evacuation, significantly impacting overall quality of life (1). The prevalence of constipation among adults in the general population is notably high, with estimates ranging from 2 to 27% (2). Patients seek relief through various therapeutic approaches, including fiber supplements, laxatives, and prescription medication. Nevertheless, approximately 50% of patients express dissatisfaction with existing treatment options, primarily due to perceived inefficacy and apprehensions regarding potential side effects (3).

Patients with constipation often attribute their symptoms to food, and targeted dietary interventions are now a cornerstone treatment (4). This dietary treatment generally recommended adequate fluid and fiber intake (e.g., whole grains, beans, greens, and fruits), specific foods (e.g., kiwifruit, prunes, aloe, and rhubarb) and dietary modification (e.g., Mediterranean diet and holistic dietary interventions) (5–8). In addition, there has been increasing research regarding the importance of the gastrointestinal microbiota to gut function (6). Homeostasis of gut microbiota and specific probiotic strains (e.g., bifidobacteria or lactobacilli) may ameliorate constipation by regulating gut motility and decreasing gut transit time (9), which indicating a promising avenue for constipation management and prevention. Furthermore, the effect of diet on gut transit time may be partly attributed to altering functionality of the gastrointestinal microbiota resulting from dietary change (10).

Nutritional intake shapes the gut microbiome, making dietary modifications a focus of interest (11, 12). Recently, Kase et al. (13) evaluated 106 studies exploring the diet-gut microbiota link in adults and identified 14 dietary elements that significantly affect the gut microbiota, either beneficially (avocado, broccoli, chickpeas, coffee, cranberries, fermented dairy, fiber, green tea, soybean and whole grains) or adversely (red meat, processed meat, refined grains, and high fat). So, they devised an innovative dietary index termed the Gut Microbiota Diet Index (DI-GM) to evaluate the dietary quality in relation to fostering a balanced gut microbiota (13). This tool could become a standardized metric for evaluating diets that promote a healthy gut microbiota balance. Nevertheless, studies investigating the association of DI-GM with constipation are lacking.

Therefore, the objective of this study was to investigate the association between DI-GM and constipation by analyzing adult data from the National Health and Nutrition Examination Survey (NHANES).

## 2 Materials and methods

### 2.1 Data source

Health data were gathered from public records spanning 3 consecutive National Health and Nutrition Examination Survey (NHANES) cycles from 2005 to 2010. This article was written in accordance with the observational clinical research STROBE guidelines. Detailed information is presented in Supplementary Table 3. The Institutional Review Board of the National Center for Health Statistics (NCHS) granted approval for the NHANES study protocol, with all participants providing consent. NHANES employs a complex, multistage probability sampling design for data collection and research methods, ensuring the gathering of extensive and dependable health information.

### 2.2 Study design and population

**FIGURE 1:**
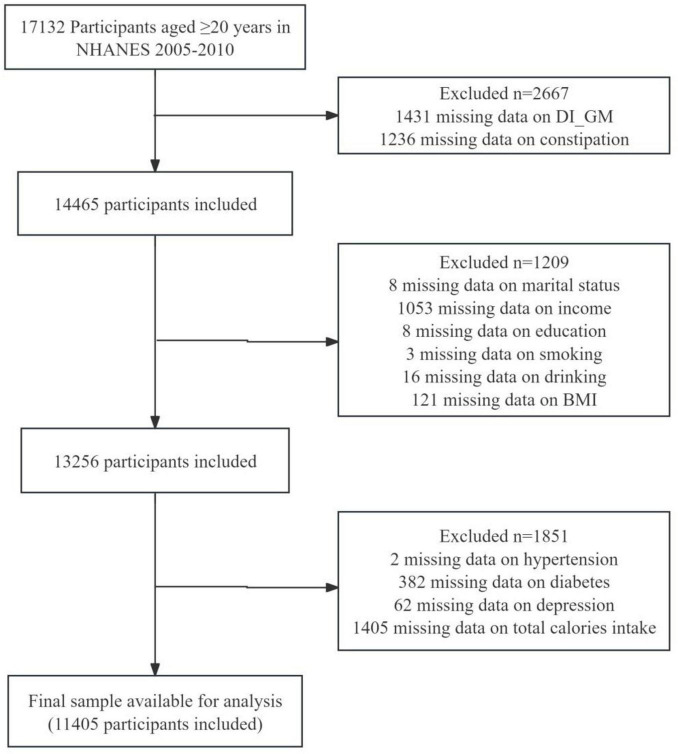
Flow chart of the screening of the NHANES 2005–2010 participants.

Our study involved a total of 17,132 participants aged ≥ 20 years from 2005 to 2010. The actual data included in our analysis covers the years 2005 to 2010, as the questionnaire data pertaining to constipation was only available during this period. Exclusion criteria of the analysis involved the absence of constipation data (*n* = 1,236), lack of DI-GM components (*n* = 1,431), and total calories intake data (*n* = 1,405), missing demographic information (*n* = 1,209), including marital status, poverty income ratio (PIR), education level, smoking, drinking status, BMI, missing comorbid conditions (*n* = 446), including hypertension, diabetes mellitus (DM), depression. The final analysis comprised 11,405 eligible participants, as depicted in Figure 1.

### 2.3 Diagnosis of constipation

In accordance with the Rome IV criteria for constipation as delineated by Mearin et al., (14) NHANES utilized participant-reported defecation frequency and stool consistency to quantify constipation among those completing the bowel health questionnaire (15). Based on NHANES data, defecation frequency was used to define constipation since stool frequency and consistency were poorly correlated (16). During the survey, participants were asked to estimate their weekly bowel movement frequency. Based on responses, participants were categorized as constipated if they reported < 3 bowel movements per week, normal if they had 3–21 movements per week, and experiencing diarrhea if they had > 21 movements weekly, aligning with previous NHANES classifications (17, 18).

### 2.4 Assessment of dietary index for gut microbiota

In accordance with the scoring criteria outlined by Kase et al., (13) the DI-GM comprises 14 food items or nutrients, which include avocado, broccoli, chickpeas, coffee, cranberries, fermented dairy, fiber, green tea (not included due to NHANES not capturing specific tea types), soybean, and whole grains as beneficial elements. Probiotics and probiotics are widely used to prepare fermented dairy products such as yogurt, cheese, kefir or freeze-dried cultures (19). Fermented dairy products containing both probiotics and prebiotics (such as lactobacilli, bifidobacteria, plantarum ST-III and inulin) will improve bowel function and constipation, which benefit people of all ages (20–22). Conversely, red meat, processed meat, refined grains, and diets high in fat (≥ 40% of total energy intake) were identified as detrimental components (13). Previous studies have shown that fatty acids are closely related to both gut microbiome and gut function. A high dietary saturated fat intake is associated with significant increase in the prevalence of constipation (23, 24). However, some polyunsaturated fatty acids (Omega-3 fatty acids or *n*-3 fatty acid) may be protective factors for constipation (25, 26). The dietary recall data from NHANES between 2005 and 2010 were employed to calculate the DI-GM scores. Detailed information on the components and scoring criteria for the DI-GM is presented in Supplementary Table 1. For gut-friendly items, a score of 1 was assigned if consumption met or exceeded the sex-specific median, otherwise 0 score. For gut-unfavorably items, a score of 0 was assigned when consumption more than sex specific median or 40% (for High-fat diet), otherwise 1 score. The DI-GM scores were aggregated to yield a total score between 0 and 13, with beneficial items scoring from 0 to 9 and unfavorable items from 0 to 4. These scores were then categorized into groups: 0–3, 4, 5, and more than 6 (27).

### 2.5 Covariates

Various potential confounding variables were gathered aligning with published research findings and clinical judgment (16, 27, 28). These factors included sex (male, female), age (continuous variable in logistic regression, while in describing participant characteristics and subgroup analyses it was categorized as < 50 years, ≥ 50 years), marital status (married, never married, living with partner, other), race (Non-Hispanic White, Non-Hispanic Black, Mexican American, other Hispanic, other race), poverty income ratio (PIR) (≤ 1.30, 1.31–3.50, > 3.5), and education level (less than high school, high school or equivalent, above high school). Physical activity encompasses the time (in minutes) that participants dedicate to various activities throughout the week, including walking, biking, household chores, work-related tasks, and recreational pursuits (29). Smoking status was categorized as never, former, and now using two questions: “Have smoked at least 100 cigarettes in your life” and “Do you smoke now?” (30). Self-reported drinking status was categorized as follows: never (consumed < 12 drinks in a lifetime), former (consumed ≥ 12 drinks in one year but not in the last year, or did not drink in the last year but consumed ≥ 12 drinks in a lifetime), now (consumed ≥ 12 drinks in a lifetime and still drinking in the last year) (31). Body mass index (BMI) was determined by calculating the BMI from measured height and weight, expressed as weight divided by height squared (kg/m^2^). Total calorie intake on the sum of two days (DR1TOT and DR2TOT) were utilized for analysis.

Self-reported cardiovascular disease (CVD) history encompassed previous diagnoses of heart failure, coronary heart disease, angina, heart attack, or stroke. These variables were dichotomized based on responses of “yes” or “no.” An individual with diabetes mellitus (DM) must have a physician’s diagnosis, including glycohemoglobin (HbA1c) levels > 6.5%, random or two-hour blood glucose levels are higher than 11.1 mmol/L in OGTTs, fasting glucose levels ≥ 7.0 mmol/L or the use of diabetes medication/insulin (32). To compute the mean blood pressure, diastolic readings of zero were omitted unless all diastolic readings were zero. If only one reading was available, it was taken as the average. In cases of multiple readings, the first reading was excluded from the calculation (33). The conditions that define hypertension are an elevated systolic or diastolic blood pressure of 140 or 90 mmHg, use of hypertensive medication and previous notification of hypertension. Hypertension is defined as one of three conditions. Within NHANES, depression screening utilized the PHQ-9 questionnaire administered by trained interviewers. A depression diagnosis was assigned if the PHQ-9 score reached 10 or higher (34). Depression status was categorized as either present (Yes) or absent (No) based on a PHQ-9 score of 10 or higher (35).

### 2.6 Statistical analysis

In adherence to NHANES analytical guidelines, our study accounted for the complex sampling design and incorporated Mobile Examination Center exam weights. Further details on the weighted analysis methodology are provided in the Supplementary Methods. We outlined characteristics associated with constipation. Continuous variables were expressed as means with standard errors (SE), and categorical variables were reported as counts and percentages (%). The Chi-squared test with Rao & Scott’s second-order correction was utilized for categorical data analysis, and the Wilcoxon rank-sum test adapted for complex survey samples was applied to continuous variables to assess significant differences.

We utilized multivariable weighted logistic regression models to investigate the association between DI-GM and constipation. Model 1 was the crude model, not accounting for any covariates. Model 2 was adjusted for age, sex, race, marital status, PIR, education level. Model 3 retained the adjustments of Model 2 and physical activity, smoking, drinking status, BMI, total calories intake. Model 4 was adjusted similarly to Model 3, with additional adjustments for CVD, hypertension, DM, depression. Logistic regression analysis was employed to ascertain the odds ratios (ORs) and 95 percent confidence intervals (95% CIs) concerning the association between DI-GM and constipation. Furthermore, we constructed multivariate-adjusted restricted cubic spline (RCS) analysis with 3 knots to fit curves and assess the potential non-linear dose-response association between DI-GM and constipation. The median DI-GM score was selected as the cutoff value. A two-piecewise logistic regression model was developed to assess the relationship between DI-GM and constipation, with adjustment for potential confounders included in model 4.

Sensitivity analyses included subgroup analyses, multiple imputation. In order to determine whether the relationship between DI-GM and constipation was stable across populations, interaction and subgroup analyses were performed according to sex (male or female), age (< 50 years or ≥ 50 years), CVD (yes or no), DM (yes or no), hypertension (yes or no), and depression (yes or no). Heterogeneity and interactions between subgroups were assessed using logistic regression models and likelihood ratio testing, respectively. In addition, to mitigate the impact of missing variables on the results, missing values were imputed using multiple imputation by chained equations, resulting in 5 imputed datasets based on variables in the final statistical model. Detailed information on multiple imputation is available in Supplementary Methods.

The statistical analyses were conducted using R software (version 4.2.1, The R Foundation for Statistical Computing, Vienna, Austria) and Free Statistics software version 2.0 (Beijing FreeClinical Medical Technology Co.,Ltd.). Two-tailed tests were employed, and statistical significance was defined as *p* < 0.05.

## 3 Results

### 3.1 Baseline characteristics

Table 1 presents the characteristics of a sample representing 157.02 million US adults with a mean age of 47.35 years (SE, 0.36), of whom 5.12 million were identified as constipation. Notably, individuals with constipation were more likely to be younger, female, Non-Hispanic White, have lower incomes and calories intake, have higher educational attainment, spend less time in physical activity, be current smokers, have lower DI-GM.

**TABLE 1:** Characteristics among adults aged 20 years or older by constipation.

Variables	Overall	Without constipation	Constipation	*P*-value
**Weighted population, *n* (in millions)**	157.02	151.89	5.12	
**Age, mean (SE), year**	47.35 (0.36)	47.47 (0.36)	43.80 (1.01)	< 0.001
**Sex, *n* (in million), %**				< 0.001
Male	75.96 (48.38)	75.24 (49.53)	0.72 (14.14)	
Female	81.05 (51.62)	76.66 (50.47)	4.40 (85.86)	
**Race, *n* (in million), %**				< 0.001
Non-Hispanic White	115.28 (73.42)	111.83 (73.63)	3.45 (67.28)	
Non-Hispanic Black	16.20 (10.32)	15.14 (9.97)	1.06 (20.67)	
Mexican American	11.51 (7.33)	11.27 (7.42)	0.24 (4.81)	
Other Hispanic	6.21 (3.96)	6.03 (3.97)	0.18 (3.59)	
Other Race	7.81 (4.98)	7.62 (5.02)	0.19 (3.65)	
**Marital Status, *n* (in million), %**				< 0.001
Married	92.14 (58.68)	89.74 (59.08)	2.40 (46.78)	
Never married	24.76 (15.77)	23.92 (15.75)	0.84 (16.48)	
Living with partner	11.58 (7.37)	11.00 (7.24)	0.58 (11.33)	
Other	28.53 (18.17)	27.23 (17.93)	1.30 (25.40)	
**PIR, *n* (in million), %**				< 0.001
≤ 1.3	28.90 (18.41)	27.14 (17.87)	1.76 (34.33)	
1.31–3.5	56.97 (36.28)	55.24 (36.37)	1.73 (33.73)	
> 3.5	71.14 (45.31)	69.51 (45.76)	1.64 (31.94)	
**Education level, *n* (in million),%**				
Less than high school	26.44 (16.84)	25.34 (16.69)	1.10 (21.46)	0.002
High school or equivalent	38.06 (24.24)	36.52 (24.04)	1.54 (30.15)	
Above high school	92.51 (58.92)	90.03 (59.27)	2.48 (48.39)	
**Smoke status, *n* (in million),%**				0.001
Never	83.16 (52.97)	80.54 (53.02)	2.63 (51.26)	
Former	40.30 (25.67)	39.36 (25.91)	0.94 (18.45)	
Now	33.55 (21.37)	32.00 (21.07)	1.55 (30.29)	
**Drinking status, *n* (in million), %**				0.023
Never	16.32 (10.40)	15.77 (10.38)	0.55 (10.86)	
Former	26.42 (16.82)	25.25 (16.62)	1.16 (22.72)	
Now	114.28 (72.78)	110.87 (72.99)	3.40 (66.42)	
**Physical activity, mean (SE), min/week**	664.48 (21.45)	668.04 (22.20)	558.88 (69.66)	0.149
**BMI, mean (SE), kg/m^2^**	28.77 (0.12)	28.80 (0.12)	28.02 (0.34)	0.027
**CVD, *n* (in million), %**				< 0.001
No	143.62 (91.47)	139.19 (91.64)	4.43 (86.36)	
Yes	13.40 (8.53)	12.70 (8.36)	0.70 (13.64)	
**Hypertension, *n* (in million), %**				0.157
No	98.74 (62.89)	95.32 (62.76)	3.42 (66.72)	
Yes	58.27 (37.11)	56.57 (37.24)	1.71 (33.28)	
**DM, *n* (in million), %**				0.535
No	136.73 (87.08)	132.20 (87.04)	4.53 (88.35)	
Yes	20.29 (12.92)	19.69 (12.96)	0.60 (11.65)	
**Depression, *n* (in million), %**				
No	146.03 (93.01)	141.97 (93.47)	4.06 (79.29)	< 0.001
Yes	10.98 (6.99)	9.92 (6.53)	1.06 (20.71)	
Total calories intake, mean (SE), kcal	4,244.74 (27.71)	4,267.26 (27.44)	3,577.32 (80.19)	< 0.001
DI-GM, mean (SE)	4.55 (0.03)	4.57 (0.03)	4.05 (0.08)	< 0.001
**DI-GM, *n* (in million), %**				< 0.001
0–3	39.38 (25.08)	37.49 (24.68)	1.89 (36.96)	
4	39.63 (25.24)	38.32 (25.23)	1.31 (25.65)	
5	36.65 (23.34)	35.60 (23.44)	1.05 (20.43)	
≥ 6	41.35 (26.33)	40.48 (26.65)	0.87 (16.96)	
Beneficial to gut microbiota, mean (SE)	2.28 (0.03)	2.29 (0.03)	1.76 (0.07)	< 0.001
Unfavorable to gut microbiota, mean (SE)	2.28 (0.02)	2.28 (0.02)	2.29 (0.06)	0.833

All means and SEs for continuous variables and percentages for categorical variables were weighted. The DI-GM ranges from 0 to 13 (including beneficial to gut microbiota [ranges from 0 to 9] and unfavorable to gut microbiota [ranges from 0 to 4]) and grouped according to 0–3, 4, 5, and ≥ 6. PIR, poverty income ratio; BMI, body mass index; CVD, cardiovascular disease; DM, diabetes mellitus; DI-GM, dietary index for gut microbiota; SE, standard error; NHANES, National Health and Nutrition Examination Survey.

### 3.2 Association between DI-GM and constipation

It demonstrates that for each one-point increment in the DI-GM, there was a 20% reduction in the prevalence of constipation in Table 2 (OR = 0.80, 95% CI = 0.74, 0.87). After adjusting for all covariates, there was an 18% drop in the rate of constipation by each point rise in the DI-GM score (OR = 0.82, 95% CI = 0.75, 0.90). Furthermore, 52% lower risk of constipation when DI-GM ≥ 6 (OR = 0.48, 95% CI = 0.33, 0.71). Both intervals indicates that the reduction in odds is statistically significant and clinically meaningful, as it suggests a consistent trend across the study population. The improvements in DI-GM scores could potentially lead to a decreased likelihood of experiencing constipation, which is an important consideration on the care and treatment planning. Additionally, the prevalence of constipation decreased significantly as the beneficial to gut microbiota increased (OR = 0.77, 95% CI = 0.67, 0.89), while the association between the unfavorable to gut microbiota and constipation was not significant (OR = 0.89, 95% CI = 0.77, 1.01, *p* = 0.074). In addition, after multiple imputation, the associations between DI-GM and constipation (crude model: OR = 0.81, 95% CI = 0.76, 0.86; adjusted model: OR = 0.83, 95% CI = 0.77, 0.89) and DI-GM ≥ 6 (adjusted model: OR = 0.5, 95% CI = 0.37, 0.69) remained significant (Supplementary Table 2).

**TABLE 2:** Multivariate regression analysis of the association between DI-GM and constipation.

Variable	Model 1^a^		Model 2^b^		Model 3^c^		Model 4^d^	
	**OR (95% CI)**	***P*-value**	**OR (95% CI)**	***P*-value**	**OR (95% CI)**	***P*-value**	**OR (95% CI)**	***P*-value**
DI-GM	0.80 (0.74, 0.87)	< 0.001	0.83 (0.76, 0.91)	< 0.001	0.82 (0.75, 0.90)	< 0.001	0.82 (0.75, 0.90)	< 0.001
**DI-GM group^e^**								
0–3	1 (Reference)		1 (Reference)		1 (Reference)		1 (Reference)	
4	0.68 (0.50, 0.93)	0.017	0.72 (0.53, 0.99)	0.043	0.70 (0.51, 0.97)	0.034	0.69 (0.50, 0.96)	0.027
5	0.58 (0.43, 0.79)	< 0.001	0.66 (0.47, 0.91)	0.014	0.63 (0.45, 0.89)	0.01	0.63 (0.45, 0.87)	0.007
≥ 6	0.42 (0.30, 0.60)	< 0.001	0.49 (0.34, 0.72)	< 0.001	0.48 (0.32, 0.70)	< 0.001	0.48 (0.33, 0.71)	< 0.001
Tend test		< 0.001		< 0.001		< 0.001		< 0.001
Beneficial to gut microbiota	0.69 (0.62, 0.76)	< 0.001	0.75 (0.66, 0.85)	< 0.001	0.76 (0.66, 0.87)	< 0.001	0.77 (0.67, 0.89)	< 0.001
Unfavorable to gut microbiota	1.01 (0.90, 1.14)	0.834	0.97 (0.86, 1.09)	0.587	0.90 (0.78, 1.02)	0.104	0.89 (0.77, 1.01)	0.074

DI-GM, dietary index for gut microbiota; OR, odd ratio; CI, confidence interval.

*^a^*Model 1: Unadjusted for any covariates.

*^b^*Model 2: Adjusted for age+sex+race+marital status+PIR+education level.

*^c^*Model 3: Model 2+smoking status+drinking status+physical activity time+BMI+total calories intake.

*^d^*Model 4: Model 3+CVD history+Hypertension+DM+depression.

*^e^*The DI-GM ranges from 0 to 11 (including beneficial to gut microbiota [ranges from 0 to 7] and unfavorable to gut microbiota [ranges from 0 to 4]) and grouped according to 0–3, 4, 5, and ≥ 6.

**FIGURE 2:**
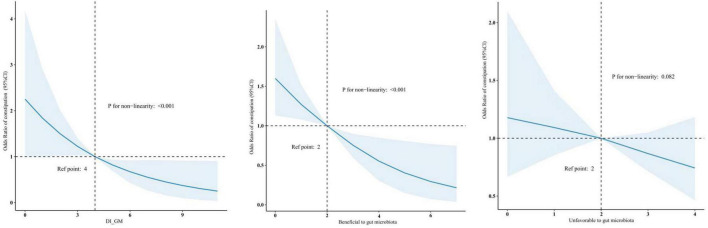
Association between DI-GM and constipation in NHANES 2005–2010 participants by RCS. CI, confidence interval; DI-GM, dietary index for gut microbiota; NHANES, National Health and Nutrition Examination Survey; RCS, restricted cubic spline.

**FIGURE 3:**
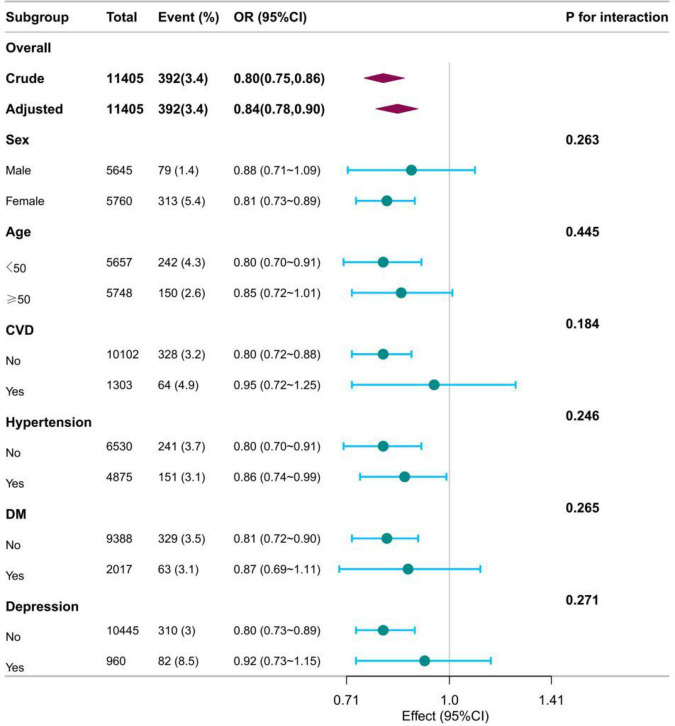
The relationship between DI_GM and constipation according to basic features. OR, odd ratio; CI, confidence interval; CVD, cardiovascular disease; DM, diabetes mellitus; Except for the stratification components itself, each stratification factor was adjusted for all other variables (age, sex, race, marital status, poverty income ratio, education level, smoking status, drinking status, physical activity time, BMI, total calories intake, cardiovascular disease, Hypertension, diabetes mellitus, depression).

The RCS showed that both DI-GM and beneficial to gut microbiota were nonlinearly associated with constipation (*P* for non-linearity < 0.001), whereas unfavorable (*P* for non-linearity = 0.082) to gut microbiota were linearly associated with constipation in Figure 2. In the two-piecewise regression models, the adjusted OR of developing constipation was 0.79 (95% CI, 0.66–0.95; *P* = 0.01) in participants with DI-GM score ≤ 4, whereas there was no association between DI-GM and constipation in participants with a DI-GM score > 4 (Table 3). Subgroup analyses were conducted, as shown in Figure 3. Stratification by sex, age, CVD, hypertension, DM, and depression did not reveal any statistically significant interactions (all *p* > 0.05). We found that the association between DI-GM and constipation was relatively stable in every subgroup.

**TABLE 3:** Association between DI-GM and constipation using two-piecewise regression models.

	Crude model		Adjusted model*	
**DI-GM**	**OR (95% CI)**	***P*-value**	**OR (95% CI)**	***P*-value**
≤ 4	0.79 (0.66, 0.94)	0.01	0.79 (0.66, 0.95)	0.01
> 4	0.75 (0.60, 0.93)	0.01	0.80 (0.61, 1.05)	0.1

DI-GM, dietary index for gut microbiota; OR, odd ratio; CI, confidence interval. *Adjusted for age, sex, race, marital status, poverty income ratio, education level, smoking status, drinking status, physical activity time, BMI, total calories intake, cardiovascular disease, Hypertension, diabetes mellitus, depression.

## 4 Discussion

Our research revealed that higher DI-GM scores, particularly those in the DI-GM ≥ 6 group and those beneficial to gut microbiota, were associated with a lower prevalence of constipation. RCS showed that the DI-GM and beneficial to gut microbiota were non-linearly associated with constipation, whereas unfavorable to gut microbiota was linearly associated with constipation. Additionally, the association between DI-GM and constipation remained stable in sensitivity analysis and subgroup analyses.

Different foods and dietary patterns are associated with the development of constipation (8). Historically, the impact of diet on gut microbiota and its potential influence on constipation has been well-documented. For example, dietary fibers, fermented dairy products, and fruits rich in probiotics (considered beneficial components of the DI-GM) may alleviate constipation by enhancing the gut microbiome (36). Probiotics to improve constipation symptoms are affected by the dose, duration of administration, and species (37, 38). Probiotics administered at higher concentrations (≥ 1,010 CFU), over extended periods, and with a variety of strains (39, 40), demonstrated greater efficacy. A systematic review showed that each extra gram of daily wheat fiber intake reduced transit time by 0.78 h in a dose-dependent way (41). In addition, compared with fermentable fiber, non-fermentable fiber (cereals, wheat bran, celery) has a better effect on stool weight increase (42). A dietary pattern according to highly intake of refined grains, red meat, processed meat, high-sugar food and high-fat dairy products, categorized as unfavorable to gut microbiota within the DI-GM, is a staple of the Western diet. The Western diet (high fat/high sugar) was found to lead to intestinal dysbiosis, increase E. coli populations, and alter host barrier function to promote intestinal colonization and inflammation by AIEC bacteria (43, 44). At the same time, the gut microbiota may modulate the inflammatory response in the hypothalamus by influencing the secretion and action of GLP-1. This mechanism alleviates metabolic disorders such as obesity, diabetes, and intestinal dysfunction by reducing hypothalamic inflammation induced by the Western diet (45). Such diet was prone to gut dysbiosis (46). The reason may be due to this dietary pattern increases harmful bacteria and decreases beneficial bacteria (47). A randomized trial indicated that replacing refined grains with whole grains could increase stool weight and frequency, and it may have a modestly positive impact on gut microbiota (48). Due to the common lifestyle characterized by high sugar and fat intake, constipation is estimated to affect 20% or more of the population, significantly impacting the quality of life across all ages and genders (49). Furthermore, a diet high in fat and sugar but low in fiber, fruits, and vegetables correlates with chronic inflammation. This inflammation is a significant mechanism linking poor dietary habits to constipation and inflammatory bowel diseases (50).

Constipation and gut microbiota are interactive. The majority of gut microbes form an intricate microenvironment, with an estimated 10 to 100 trillion symbiotic bacteria per person, closely interacting with the host and influencing health and disease (51, 52). Constipation can trigger a systemic inflammatory response that reduces gut microbiota diversity and leads to gut ecological imbalance. An experimental study found that constipated mice had thicker muscle layers, higher levels of cytokines like IL-17 and IL-23, and lower IL-22 levels (53). Intestinal flora can influence innate defense responses and intestinal epithelial homeostasis by modulating TLR signaling, which in turn affects immune activation (54). Specific gut flora (ruminiclostridium or intestinibacter) can affect innate defense responses and intestinal epithelial cell homeostasis by regulating TLR signaling, which in turn affects immune activation (55). Moreover, recent research indicates that Western dietary patterns may disrupt the gut microbial ecosystem and induce chronic intestinal inflammation (56). This situation is further worsened by the synergistic effects between constipation and inflammation, particularly in constipation patients who also suffer from depression (57). So, diet is closely related to gut microbiota and constipation, and it is one of the most common and simplest treatment options. Our study shows that DI-GM is negatively associated with the lower risk of constipation and highlights the importance of maintaining a healthy dietary pattern.

To our knowledge, this is the inaugural study to investigate the link between DI-GM, a dietary quality index correlating with gut microbiota diversity, and constipation. Thanks to the stringent quality control measures and advanced sampling designs employed by NHANES, we were able to assess the association in a substantial and varied adult population across the United States. Moreover, sensitivity analyses, including multivariable weighted logistic regression and subgroup analyses, bolstered the robustness and reliability of our results.

Our study faces limitations. Firstly, its cross-sectional design prevents us from determining causation between DI-GM and constipation. Reverse causality also cannot be ruled out. More prospective or randomized controlled studies are necessary to confirm the underlying mechanisms between DI-GM and constipation. Secondly, like many studies, we cannot fully eliminate the potential for confounding effects due to unmeasured variables or unknown confounders that might introduce measurement error. Thirdly, although the original DI-GM incorporated 14 foods, the NHANES 24-h dietary recall data did not capture specific tea consumption types, making it impossible to include these parameters. Fourthly, the use of self-reported 24-h dietary records to assess DI-GM and a bowel health questionnaire for constipation could introduce recall bias, and some covariates relied on self-reporting as well. To minimize bias, we have used the mean of 24-h recalls, multivariable weighted logistic analysis. Fifthly, the DI-GM comprises numerous fiber-rich foods, which complicates the distinction between the effects of fiber and microbiota changes on chronic constipation. Future studies could design more precise experiments to individually assess the effects of fiber and other dietary components on the gut microbiota and constipation symptoms. Lastly, Bowel Health Questionnaire data were only collected in NHANES between 2005 and 2010. This prevented us from using NHANES data from different time periods (especially recent years) for further validation. More prospective studies are needed in the future to further validate our findings.

## 5 Conclusion

The DI-GM, a novel dietary quality index linked to gut microbiota diversity, showed a negative correlation with constipation rates. We recommend to adopt a diverse plant-based diet rich in fiber, probiotics, and prebiotics, while reducing red meat, processed foods, and high-fat intake to promote a healthy gut microbiota. Considering the strong association between diet, gut flora, and constipation, future studies integrating dietary interventions based on the DI-GM are essential in mitigating the prevalence of constipation.

## Data Availability

The datasets presented in this study can be found in online repositories. The names of the repository/repositories and accession number(s) can be found in this article/Supplementary material.
